# Deep Resequencing of 9 Candidate Genes Identifies a Role for ARAP1 and IGF2BP2 in Modulating Insulin Secretion Adjusted for Insulin Resistance in Obese Southern Europeans

**DOI:** 10.3390/ijms23031221

**Published:** 2022-01-22

**Authors:** Diego Bailetti, Federica Sentinelli, Sabrina Prudente, Flavia Agata Cimini, Ilaria Barchetta, Maria Totaro, Alessia Di Costanzo, Arcangelo Barbonetti, Frida Leonetti, Maria Gisella Cavallo, Marco Giorgio Baroni

**Affiliations:** 1 Department of Clinical Medicine, Public Health, Life and Environmental Sciences (MeSVA), University of L’Aquila, 67100 L’Aquila, Italy; federica.sentinelli@uniroma1.it (F.S.); maria.totaro@univaq.it (M.T.); arcangelo.barbonetti@univaq.it (A.B.); 2 Department of Experimental Medicine, Sapienza University of Rome, 00185 Rome, Italy; flavia.cimini@yahoo.it (F.A.C.); ilaria.barchetta@uniroma1.it (I.B.); gisella.cavallo@uniroma1.it (M.G.C.); 3 Research Unit of Metabolic and Cardiovascular Diseases, Fondazione IRCCS Casa Sollievo della Sofferenza, 71013 San Giovanni Rotondo, Italy; s.prudente@css-mendel.it; 4 Department of Translational and Precision Medicine, Sapienza University of Rome, 00185 Rome, Italy; alessia.dicostanzo@uniroma1.it; 5 Diabetes Unit, Department of Medical-Surgical Sciences and Biotechnologies, Santa Maria Goretti Hospital, Sapienza University of Rome, 04100 Latina, Italy; frida.leonetti@uniroma1.it; 6 Neuroendocrinology and Metabolic Diseases, IRCCS Neuromed, 86077 Pozzilli, Italy

**Keywords:** diabetes, disposition index, obesity, next-generation sequencing, targeted resequencing, extremes, insulin secretion, insulin resistance

## Abstract

Type 2 diabetes is characterized by impairment in insulin secretion, with an established genetic contribution. We aimed to evaluate common and low-frequency (1–5%) variants in nine genes strongly associated with insulin secretion by targeted sequencing in subjects selected from the extremes of insulin release measured by the disposition index. Collapsing data by gene and/or function, the association between disposition index and nonsense variants were significant, also after adjustment for confounding factors (OR = 0.25, 95% CI = 0.11–0.59, *p* = 0.001). Evaluating variants individually, three novel variants in *ARAP1, IGF2BP2* and *GCK*, out of eight reaching significance singularly, remained associated after adjustment. Constructing a genetic risk model combining the effects of the three variants, only carriers of the *ARAP1* and *IGF2BP2* variants were significantly associated with a reduced probability to be in the lower, worst, extreme of insulin secretion (OR = 0.223, 95% CI = 0.105–0.473, *p* < 0.001). Observing a high number of normal glucose tolerance between carriers, a regression posthoc analysis was performed. Carriers of genetic risk model variants had higher probability to be normoglycemic, also after adjustment (OR = 2.411, 95% CI = 1.136–5.116, *p* = 0.022). Thus, in our southern European cohort, nonsense variants in all nine candidate genes showed association with better insulin secretion adjusted for insulin resistance, and we established the role of *ARAP1* and *IGF2BP2* in modulating insulin secretion.

## 1. Introduction

Type 2 diabetes (T2D) is a complex disease affecting the world’s population at epidemic rates and whose pathophysiology remains elusive. T2D is projected to affect up to 700 million people worldwide by 2045, with a 51% global increment [1]. The burden of diabetes will affect mostly developing countries, as diabetes is strongly associated with urbanization and increased wealth. T2D carries an increased risk of developing a wide range of macrovascular (cardiovascular, cerebrovascular and peripheral artery diseases) and microvascular complications (retinopathy, neuropathy and nephropathy) [2], with large differences in prevalence, severity and comorbidities across global populations. T2D is characterized by an inadequate β-cell response to the progressive insulin resistance that accompanies advancing age, inactivity and weight gain [3]. Impaired insulin secretion is considered one of the first defects leading to impaired glucose metabolism and the development of T2D. A genetic contribution is well recognized in diverse forms of both early-onset and adult-onset diabetes [4,5,6,7]. Aside from genetic determinants, several other factors are involved in the pathogenesis of diabetes, obesity and diabesity (co-occurrence of obesity and diabetes). For example, studies pointing to the involvement of oxidative stress show that it is significantly higher in obese vs. nonobese subjects. It is positively correlates with worse clinical measurements (e.g., BMI, waist, fasting plasma glucose, total cholesterol, etc.). Furthermore, reactive oxygen species (ROS) levels are higher in obese diabetic vs. nondiabetic obese subjects. On the other hand, the measurement of antioxidant levels negatively correlates with BMI and total cholesterol [8]. Additionally, there are well-established prenatal and risk factors that influence metabolic impairment onset and development, such as those reported for gestational diabetes mellitus [9]. Glucose metabolism and T2D are well-established multifactorial processes. To deconstruct the heterogeneity of T2D, cluster analyses using serum biomarkers and clinical data of individuals have been performed to identify T2D subgroups [10,11]. Cluster analyses have been used to change the current paradigm of classifying patients with diabetes mellitus. This analysis comprises an unbiased cluster allocation using common variables such as autoimmunity, age at diagnosis, BMI, glycemic control and homeostasis model estimates of β-cell function and insulin resistance. The final scope is a refined classification that could provide a powerful tool to individualize treatments and identify individuals with increased risk of complications at diagnosis [10,11]. These studies suggest newer routes for future research, but there are also limitations given the nature of the variables included in the analyses, which are bound to change over time. Contrariwise to serum biomarkers, germline genetic variants associated with T2D remain constant, regardless of age, disease state or treatment, and are more likely to identify T2D causal mechanisms. In the last decade, large-scale genomic studies, including genome-wide association studies (GWAS), have identified over 400 common variants in more than 100 loci that confer disease susceptibility [12,13]. Despite the great number of loci linked, there is an extensive gap between the discovery of many T2D-associated single-nucleotide polymorphisms (SNPs) and the understanding of their physiological impact on T2D pathogenesis or their clinical use as risk factors. Furthermore, a consistent discrepancy between observed heritability and recognized genetic background in complex disease is well established, often reported as “missing heritability” [14,15]. Identification of genetic factors and genes that underlie T2D could shed light on diabetes molecular background and inform clinical management strategies, including patient stratification, personalized medicine or optimization of study design of randomized controlled trials.

The genes identified so far are mainly associated with pancreatic β-cells maturation or function [16], but all the mechanisms affecting β-cell function have not yet been fully understood.

Common variants associated with complex disease by GWAS cause modest increases in disease risk, with odds ratios generally <1.2 [17,18,19,20,21]. They may indicate a gene or a locus strongly involved in that disease, as most GWA studies use technologies that allow investigating only known or common mutations. As suggested by Rivas et al., targeted resequencing may help to discover other new variants, especially rare or low frequencies, harbored in genes that may exert extra influence on the trait or disease [22,23]. Furthermore, it has been reported several times that multiple rare variants can have a stronger effect on complex traits than individual common variants [24,25].

It is therefore clear that there is an increasing need to analyze in-depth candidate genes by direct sequencing [25,26,27]. This strategic approach is now made possible thanks to next-generation sequencing (NGS) technologies. Moreover, a new powerful strategy was proposed to increase the chance to highlight causal variants involved in modulating a phenotype in association studies. It consists of sequencing samples from the extremes of a trait [28]. Intuitively, such samples should be enriched for the burden of alleles influencing a trait, thus improving statistical power to discover risk/protective variants and association to the trait [24,29,30]. Additionally, it allows for more homogeneity into cohorts, determining a greater power to detect association and helping to identify markers with higher ORs [31].

Given the fact that β-cell function and glucose-stimulated insulin secretion appear to be the traits most strongly associated with T2D pathogenesis, and that insulin resistance is accompanied by early compensatory upregulation of insulin secretion, the best method for measuring β-cell function is evaluating insulin secretion adjusted for insulin sensitivity. This ratio, called the disposition index (DI), assumes that at the same glucose tolerance this index remains constant. DI is calculated by insulin secretion/insulin resistance (ΔI/ΔG ÷ IR), with several formulas being proposed depending on available measurements [32,33]. The loss of function of β-cells, which reduces their capacity to raise insulin secretion to compensate for insulin resistance, results in a lower DI [34,35,36]. Thus, DI can be considered a measure of the functionality of the pancreas and can predict the normal β-cell response adequate for any degree of insulin resistance. In diabetes, β-cells are unable to respond adequately to insulin resistance, thus determining the appearance of impaired glucose regulation and altering the disposition index. DI avoids using gold-standard techniques (i.e., euglycemic clamp), which is difficult to apply on a wide scale [34,35,36].

Thus, we aimed to evaluate common and low-frequency (1–5%) variants in genes associated with insulin secretion by targeted resequencing in subjects selected from the extremes of insulin release measured by DI. We selected the nine genes most associated in the literature to β-cell’s insulin secretion who reach genome-wide significant association with T2D from published GWAS (*p* ≤ 5 × 10^−8^). Genes selected are *ADAMTS9, ADCY5, CDAKL1, IGF2BP2, JAZF1, GCK, NAT2, KCNQ1* and *ARAP1*. These genes are all involved in the process of insulin secretion, including synthesis, trafficking, rate, localization and vesicles formation. Published odds ratios (ORs) along with 95% confidence intervals (CIs) and references for selected genes are shown in Supplementary Table S1 [37,38].

To our knowledge, no studies involving NGS of such candidate genes have been performed so far in obesity and diabetes associated with obesity, and no deep resequencing has been implemented on these selected genes.

## 2. Results

### 2.1. Study Samples and Quintiles of Disposition Index

The clinical features of all 757 participants presented as 20% versus 80% (1st vs. 5th quintile) of the disposition index are shown in Table 1. Table 1 also shows *p*-values calculated between the extremes. Sex distribution was similar between the two quintiles: in the 20% DI (*n* = 377), 18 (31.3%) were males, and 259 (68.7%) were females; in the 80% DI (*n* = 380), 94 (24.8%) were males, and 285 (75.2%) were females.

Our cohort shows, as expected, a high prevalence of obesity, as the median BMI (Kg/m^2^) in the two extremes is 39 and 40, respectively. Additionally, the lowest and highest quintiles of DI differ significantly for most of the lipid measurements, with the lower insulin secretion (lower DI) showing the worst metabolic profile (e.g., higher LDL, lower HDL, higher TGs).

Nonparametric variables are reported as medians along with the 25° and 75° percentiles (25°% and 75°%), the 1st and 5th quintiles (Q1 and Q5), respectively. *P* was evaluated with the Student’s *t*-test if the variable was assessed parametric or the Mann–Whitney U test if nonparametric. SBP: systolic blood pressure; DBP: diastolic blood pressure; Col: cholesterol; HDL: high-density lipoprotein; LDL: low-density lipoprotein; TG: triglycerides; AST: aspartate aminotransferase; ALT: alanine aminotransferase; FPG: fasting plasma glucose; Glu 120′: PLASMA GLUCOSE after oral glucose tolerance test (OGTT) at 120′; FPI: fasting plasma insulin; Ins 120′: plasma insulin after OGTT at 120′; ISI: insulin sensitivity index; IGI: insulinogenic index; DI: disposition index; HOMA-IR: homeostatic model assessment for insulin resistance; HOMA-B: homeostatic model assessment of β-cell function; c-pep 0’: C-peptide test at 0′; HbA1c: glycosylated hemoglobin.

### 2.2. Number and Type of Variants Observed in Study Subjects

Quality assessment for sequencing data resulted in a QScore ≥ 30 for 72.7% of bases, QScore ≥ 20 for 81.7% of bases, and QScore ≥ 12 reads for 100% of bases. The fraction of the captured targeted regions (29′685 bp) covered by at least one read was 98.2%. After the sequencing runs, acquisition of data and variant calling, we observed 5636 variants in the raw dataset. Filter setup is explained in detail in the Materials and Methods section (Section 4). The first filtering retained 2751 variants in the whole sample, 1876 of which were differentially distributed in one of the extremes. After the final filtering, 1221 variants were retained. Among these, 879 affected protein function by in silico analysis. The gene distribution and function of these 1221 variants are reported in Table 2.

### 2.3. Association Analyses

After this discovery phase, binary logistic regression was performed to evaluate the association of variants, both alone and collapsed by gene or function, with DI extremes. When analyzing all variants that passed the filter (*n* = 1221), collapsed by gene and/or function, only the association between DI and nonsense variants were significant, also after adjustment for established risk factors such as age, gender and BMI. Carriers of these stop variants had overall 75% less probability to be in the lower, more pathologic, extreme of DI (OR = 0.25 95% CI = 0.11–0.59 *p* = 0.001). Analysis of goodness of fit showed that this model explained around 25% of the variable’s variance (R^2^ Nagelkerke = 0.25; Hosmer–Lemeshow test = 0.063). Thus, in our cohort, nonsense variants seem to be protective regarding insulin secretion status. We then evaluated the variants individually, and eight of them, within four genes (*ARAP1, GCK, KCNQ1* and *IGF2BP2*), were significantly associated with one of the extremes of DI. After adjustment for confounding factors (age, gender and BMI), only three novel variants, in the *ARAP1, IGF2BP2* and *GCK* genes, remained significantly associated (see Table 3). The strongest significant effect was observed for carriers of the *IGF2BP2* variant, showing an 85% (CI = 50% to 95%) reduced probability to be in the pathological extreme of insulin secretion relative to insulin resistance. Carriers of SNPs in *ARAP1* and *GCK* showed similar results in significance and effect, with a mean probability of 70% and 62%, respectively. Other genes did not reach significance in our samples.

Of note, 22 out of 29 (75%) of the carriers of the missense variant in *ARAP1* were diagnosed as normal glucose tolerant (NGT), assessed by OGTT. Additionally, between the 18 carriers of the intronic variant in *IGF2BP2*, 13 (72%) were identified as NGT.

### 2.4. Genetic Risk Model of Significant Variants

Due to the low frequency observed for the variants in the *ARAP1, IGF2BP2* and *GCK* genes, without subjects carrying more than one variant, we constructed a genetic risk model. We initially used all three associated variants. Unexpectedly, when all three SNPs in the genetic risk model were analyzed by binomial regression, *GCK* did not reach significance. We therefore used only the *ARAP1* and *IGF2BP2* variants to build a genetic model. Association analysis of this genetic risk model adjusted for age, gender and BMI, strengthened both significance and effect compared to the individual association (see Table 4). Carriers of at least one of the two allele variants showed to be, on average, 78% less likely to be in the lower, pathological, extreme of DI, with a reduction of between 90% and more than 50%. Interestingly, this model explained more than 25% of DI variance (R^2^ Nagelkerke = 0.254, *p* = 0.015).

Observing a large number of NGT between carriers of each SNP, a *post hoc* regression analysis was performed to assess whether glucose tolerance status is associated with being carriers of at least one of the two selected variants (see Supplementary Table S2). Indeed, carriers of any of the two variants in the genetic risk model had a 2.4-fold higher probability to be NGT after adjustment for age, gender and BMI than noncarriers (OR = 2.41 95% CI = 1.14–5.12 *p* = 0.02).

## 3. Discussion

In this study, we employed a candidate gene approach with NGS technology to confirm and validate results derived from genome-wide association analyses in a southern European real-life cohort with a high prevalence of overweight/obesity. As previously discussed, GWAS results should be confirmed and validated with further studies, including molecular and cellular studies or targeted sequencing [22,23]. Because of its own technique, GWA studies focus on common variants. Empirical observations showed that heritability explained by common variants emerging from GWAS is limited, especially for multifactorial diseases such as diabetes or obesity. Possibly, rare and low-frequency variants harbor more effect, and for a selective pressure in fitness, they are less frequent. Additionally, GWAS-derived variants may not directly affect the trait but might be in linkage with a “real” causal variant. Furthermore, SNPs in noncoding regions could affect protein regulation through several ways pre/post-transcriptionally and translationally, such as modulation of chromatin, RNA transcription, translation and stability. For all this evidence, we performed deep resequencing of nine genes selected from the most strongly associated with insulin secretion from GWAS (see Supplementary Table S1), to confirm the genes’ roles and to extend our knowledge on the T2D molecular mechanism.

Considering all variants together, collapsed by function or position in the nine genes, the presence of nonsense variants is associated significantly with a reduced chance to be in the lower quintile of the disposition index (i.e., the quintile with the most reduced insulin secretion adjusted for insulin resistance). Carriers of nonsense variants in this dataset showed on average a 75% reduced probability to be in the worst quintile of insulin secretion, ranging from 45% to 90%. To explain this association of the nonsense variants with a protective role, we could hypothesize that those nonfunctional forms of these proteins could interfere with molecular feedback pathways responsible for the processing of insulin secretion. For example, reducing the expected increase in insulin secretion was secondary to the rise in insulin resistance. This may result in the lowering of DI and possibly slowing down diabetes progression. However, we cannot exclude additional mechanisms, such as enhancing or blocking secondary effectors of insulin secretion or signaling or positive/negative feedback pathways.

Analyzing data individually, three variants in the *GCK, ARAP1* and *IGF2BP2* genes were significantly associated with DI. Evaluating these three variants in a single predictive model, the variant in *GCK* lost significance. Thus, its contribution to the association was dropped. The remaining associated variants were chr3:185363420 A > G in *IGF2BP2* and chr11:72422158 A > C, in *ARAP1*. Both were novel variants not previously reported.

### 3.1. IGF2BP2 Variant

chr3:185363420 A > G in *IGF2BP2* (NM_006548) is a variant in intron 15 (c.1708-9 A > G; g.184410 G > C), next to the start of exon 16/16. It is located two nucleotides apart from a rare known variant (rs1199891239 c.1708-7 G > C) laying in a splice site. IGF2BP2 binds and modulates insulin growth factor 2 (*IGF2*) 5’-UTR mRNA, affecting its localization, translation and stability. Moreover, it modulates the rate and site of translation of target transcripts and protects them from endonucleases or microRNA-mediated degradation [39]. *IGF2BP2* is highly expressed in pancreatic islets, but its contribution to diabetes is unclear. Animal models show that it is implicated in regulating growth and metabolism. The null model results in dwarf mice resistant to obesity and fatty liver if subjected to a high-fat diet (HFD). Results in tissue-specific knockout models are divergent. *IGF2BP2* knockout in β-cells showed reduced fasting insulin, c-peptide levels and lower glucose-stimulated insulin secretion in animals fed an HFD. KO *IGF2BP2* hepatocytes mice showed high resistance to fatty liver in HFD. They also show reduced fat mass and lipid oxidation. In a mouse model where IGF2BP2 was instead upregulated in the liver by the transgene, lipid storage was enhanced, which led to fibrosis and NAFLD [40,41,42]. Of note, the insulin (*INS*) gene is located near the *IGF2* gene, and the *INS-IGF2* readthrough transcript (*INSIGF*) has been observed. It aligns the *INS* gene in the 5’ region to the *IGF2* gene in the 3’ region. *INSIGF* expression was found in the placenta, liver, pancreas, fat, ovary and endothelial tissues, the tissues most strongly implicated in regulating metabolism. It has been speculated that IGF2BP2 could bind the *INSIGF* transcript with the same domain utilized with *IGF2*, also fine modulating insulin expression and consequently insulin receptor activity, thus ameliorating DI [43].

Given this scenario, we can speculate that this could be a splicing variant likely impairing IGF2BP2 protein function. Thus, our results are in line with previous studies, which demonstrated that *IGF2BP2* deletion in mice improves glucose tolerance and insulin sensitivity and protects mice from diet-induced obesity and fatty liver [44]. Finally, *IGF2BP2* also contributes to obesity and T2D through its regulation of *IGF2*, which participates in the pathogenesis of these diseases [45].

### 3.2. ARAP1 Variant

The variant in *ARAP1* (NM_001040118), chr11:72422158 A > C is a missense, Val 374 Gly (V374G). Several prediction tools (such as SIFT, PolyPhen2, MutPred, FATHMM and PROVEAN) suggest that V374G is deleterious or probably damaging for protein function. For example, PolyPhen-2 analysis of V374G results as probably damaging with a score of 0.971 (sensitivity: 0.77; specificity: 0.96). ARAP1 phosphorylates a large family of GTPases, which modulate actin and cytoskeleton through ARF and RHO proteins. It is wildly expressed and involved in the Golgi apparatus, molecular trafficking and cellular membrane function. ARAP1 is activated by (3, 4, 5) trisphosphate (PIP3) and 3.4 PI (PIP2) with less efficiency. PIP3 is a secondary signaling lipid generated by insulin signaling. It has been reported in the drosophila cell model that deleting a negative regulator for PIP3 (phosphatidylinositol 5 phosphate 4-kinase (PIP4K)) causes an increase in PIP3 levels, with enhanced insulin sensitivity [46]. Additionally, overexpression of *ARAP1* mRNA in the human pancreatic cell, due to a common functional variant (rs11603334; MAF in non-Finnish EU: 16%) in the promotor, was associated with decreased production of proinsulin and an increase in T2D risk [47]. Contrariwise, a proinsulin-raising variant was associated with lower fasting glucose (0.019 mg/dL per allele; *p* = 1.7 × 10^−4^), lower A1C (0.023%; *p* = 0.02), improved β-cell function (*p* = 1.1 × 10^−5^) and a lower risk of T2D (OR = 0.88; *p* = 7.8 × 10^−6^) [48]. Furthermore, ARAP1 regulates the ARF family of GTPases, which control several key cellular processes, including membrane trafficking such as secretion or endocytosis, ciliogenesis, lipid metabolism, energy balance, motility, division, apoptosis and transcriptional regulation [49]. Among the ARF family, ARAP1 strongly interacts with ARF6, which is a known modulator of insulin secretion [50,51,52,53]. Thus, as *ARAP1* is involved in the Golgi apparatus, molecular trafficking and cellular membrane, its contribution to insulin secretion can be postulated by its ability to affect insulin storage in vesicles, their movement, membrane binding and release. A nonfunctional ARAP1 protein may lead to a decrease in glucose-stimulated insulin secretion, possibly via ARF6. Overall, our data are in line with Kulzer and Strawbridge’s results, where higher levels of *ARAP1* mRNA are associated with an increased risk of T2D and decreased proinsulin release, while the reduction in ARAP1 levels or function ameliorates β-cell function and T2D risk.

### 3.3. Post hoc Analysis

In our results, unexpectedly, the *ARP1* and *IGF2BP2* variants protectively influence DI. Analysis showed an increased probability for carriers to be in the highest quintile of insulin secretion adjusted for insulin sensitivity. This condition is also confirmed by the high number of carriers presenting with normal glucose tolerance. Our post hoc analysis displayed a significant association between normal glucose metabolism and the variants in a genetic risk model (see Supplementary Table S2). Specifically, carriers of any variant present in the genetic risk model are significantly associated (*p* = 0.02) with a 2.4-fold (95% CI = 1.14–5.12) higher probability to be normoglycemic (NGT).

### 3.4. Novelties and Limits

As limits, our study samples allow us to detect only ORs of relative medium magnitude (1.7–2.6 depending on MAF). Thus, we could have missed some variants that exert weaker effects or that become significant only in very large datasets. However, as suggested by Nejentsev et al., in complex diseases, such as T2D, there may be no low-frequency variants, or very few, with very strong effects (e.g., allele OR >3). Even if such variants have large impacts on a certain molecule’s function, it is possible that in complex multifactorial diseases, such a molecule and its biological pathway are just one of many contributing to the pathogenesis. Nevertheless, the discovery of such rare variants using high-throughput sequencing will help to identify disease genes in the loci found by GWAS in various complex diseases. [54].

Results from published GWAS and metadata studies need to be assessed in specific and real-life populations to generalize findings in gene function and ethnicity. The novelties of this study are the targeted resequencing searching for associated variants, especially rare or low frequencies, harbored in genes identified by GWAS [22,23] in a Central Italy cohort, and the study design, exploring the extremes of a trait (the disposition index of insulin secretion), which allows more homogeneity in the study sample and enhances statistical power. Our study shows that nonsynonymous variants in nine candidate genes are all associated with better insulin secretion adjusted for insulin resistance. Additionally, from the single analysis, the novel low-frequency variants chr11:72422158 A > C in *ARAP1* and chr3:185363420 A > G in *IGF2BP2* showed significant association with healthier insulin secretion, relative to insulin sensitivity, measured by DI.

## 4. Materials and Methods

### 4.1. Study Subjects

From a cohort of 2232 white Italian patients enrolled at Policlinico Umberto I Hospital of University of Rome Sapienza, Italy, attending the outpatient clinics of Endocrinology and Diabetology during the years 2001–2018, we selected 757 subjects from the first and last quintiles of DI distribution for the sequencing study. Ethnicity was self-reported. Anthropometric and clinical measurements comprising a minimum 3-point OGTT were recorded in an anonymized database. Body mass index (BMI) was calculated as body weight (kg) divided by height squared (m^2^) and was used as a marker of obesity. Glucose tolerance status (NGT, IFG: impaired fasting glucose, IGT: impaired glucose tolerance, DM: diabetes mellitus) was diagnosed according to ADA 2021 [2]. Plasma/serum biochemistry (glucose, insulin, full lipid profile, transaminases, etc.) was measured in the same laboratory with standard techniques. The disposition index (DI), as well other clinical derivative estimates of insulin release and sensitivity, was calculated from OGTT. The insulinogenic index (IGI30), as a measure of glucose-stimulated insulin secretion and also of β-cell function, was calculated as (Ins30–Ins0)/(Glc30–Glc0) [55]. Insulin sensitivity (ISI) was estimated as [56]: 10,000/(Glc0 × Ins0 × GlcMean × InsMean)1/2. DI was calculated as the product of IGI30 and ISI for estimating insulin secretory capacity adjusted for insulin resistance [57].

Samples to be sequenced were selected from two extremes of insulin secretion calculated by DI (1st and 5th quintiles or <20% and >80% of DI distribution) in the whole cohort comprising more than 2000 patients. To compare genetic variants between subjects with a better and worse DI index of insulin secretion, they were analyzed as a case–control cohort.

Study protocols and informed consent procedures were approved by the local Institutional Ethics Committees (Protocol No.: 151/14, Ref.: 3070/30-01-2014), and all participants gave signed informed consent. The study was carried out in accordance with the Declaration of Helsinki, as revised in 2000.

### 4.2. DNA Extraction and Sequencing

DNA extraction was carried out from white-cell peripheral blood by the standard procedure of the salting-out method. DNA was then evaluated for quality (NanoDrop 2000 Spectrophotometers, Thermo Fisher, Waltham, MA, USA) and quantified (Qubit Fluorometric Quantification, Thermo Fisher, Waltham, MA, USA) before processing. The design of the gene panel, which included 9 genes (i.e., ADAMTS9 NM_182920, ADYC5 NM_183357, ARAP1 NM_001040118, CDKAL1 NM_017774, GCK NM_000162, IGF2BP2 NM_006548, JAZF1 NM_175061, NAT2 NM_000015 and KCNQ1 NM_000218), was made with DesignStudio (Illumina) online software. GC content, specificity, probe interaction and presence of SNPs were considered in the probes’ selection. The probe’s panel was optimized through in silico simulation. Then, it was tested and validated in Illumina WetLab. Each probe comprehends the sequences designed for capturing the regions of interest, including one specific sequence to be utilized in successive genetic amplification. NGS was made through Illumina TrusSeq Custom Amplicon (Illumina, San Diego, CA, USA) technology in the Illumina MiSeq (cartridge V2 300c) sequencer. Probes were designed to amplify, by 219 amplicon intermediates, the coding region, along with 100 bp flanking on both sides of the exons, for a total of 29,685 bp. The study design provides a mean coverage of 100× for each sample, allowing more accurate base calling, especially for low-frequency variants. Briefly, the sequencing process started with amplification of all exons and flanking 100 bp from selected genes via amplicon generation. They represent the genetic libraries. Obtained libraries from all samples were purified singularly through the magnetic beads approach. Then, they were normalized and pooled together to perform high-throughput parallel sequencing by cluster generation and successive sequencing by reading fluorescence. Following the runs of the libraries on a MiSeq system, data were automatically processed using built-in and on-cloud software, such as Illumina software BaseSpace (https://basespace.illumina.com, accessed on 20 February 2021). The output variant call format (VCF) file was then annotated through BaseSpace, VariantStudio (Illumina), wANNOVAR (http://wannovar.wglab.org accessed on 20 September 2021) and Cravat (https://www.cravat.us/CRAVAT/ accessed on 20 September 2021) software. Collected data were analyzed both with dedicated software and plug-ins made by Illumina and free bioinformatics and biostatistics tools (SAMtools, BCFtools, VCFtools) [58]. The variants were annotated as nonsense, missense, splicing, synonymous and UTR following published guidelines [59]. Functional affection of the variants was investigated using the major prediction programs available: SIFT, PolyphenII, SNP&go, Provean, Mutation T@ster, Mutation Assessor, FATMHH and CADD were used for exonic, while ESEfinder, GeneSplicer, and NetGene2 for intronic variants were used. This methodological approach allowed us to assess, in transcribed regions of genes associated with insulin secretion, the distribution of genetic variants within our southern European cohort.

MAF was obtained by assessing the gnomAD browser genome (https://gnomad.broadinstitute.org/ accessed on 20 November 2021) and the 1000 genome database (https://www.internationalgenome.org/ accessed on 20 November 2021).

### 4.3. Variants Filtering

SNPs and insertions/deletions were identified across the targeted subset of the reference genome (hg19). We filtered all variants observed for quality and quantity of reads, as well as information on annotated variants. Several filters were subsequently applied. First were selected data with more than 30 reads (DP > 30) and genotype quality equal to or more than 30 (GQX ≥ 30). Additionally, default sample and record levels filters were applied from Illumina VCF metrics. Then, variants with genotype quality less than 99 (GQX < 99) were filtered out to avoid most false-positive results from NGS. All passing-filter variants were retained.

### 4.4. Statistical Analysis

Categorical variables were compared with the Chi-square test or Fisher’s exact test. Differences between continuous variables were evaluated by two-tailed Student’s *t*-test and ANOVA. For nonparametric measures, the Mann–Whitney U test was used. To control for the effects of other confounding factors, multivariate linear and logistic regression analyses were performed. The adequacy of the final model was assessed using the Hosmer–Lemeshow goodness-of-fit test. Furthermore, the Nagelkerke R^2^ was calculated to indicate how useful the explanatory variables in the model were in predicting DI association [60]. Variants in carriers were considered both individually and collapsed together, evaluating the possible combined effect. In general, *p* < 0.05 was taken as statistically significant, except where Bonferroni correction was applied. All statistical analyses were performed with SPSS (IBM Corp. Released 2017. IBM SPSS Statistics for Windows, Version 25.0. Armonk, NY, USA.), R and Rstudio (Rstudio Team (2020). Rstudio: Integrated Development for R. Rstudio, PBC, Boston, MA, USA. http://www.rstudio.com/ accessed on 20 February 2021).

### 4.5. Power Calculation

According to Guey et al. [29], we estimated that an ascertainment sample of 757 individuals in the top and bottom quintiles of a quantitative trait (DI in our study), assuming a disease prevalence of 0.15, gave a power >80% with a significance level of 0.05, to detect low-frequency (1–5%) variants, with ORs ranging between 1.7 and 2.6, depending on allele frequency (from 1 to 5%), from a population of more than 2000 subjects. Power analysis was performed by the Genetic Association Study (GAS) Power Calculator (© 2017 Jennifer Li Johnson, University of Michigan; available online: https://csg.sph.umich.edu/abecasis/gas_power_calculator/index.html accessed on 20 November 2021).

## 5. Conclusions

In conclusion, nonsense variants in all nine candidate genes showed association with better insulin secretion adjusted for insulin resistance. Furthermore, in the *ARAP1* and *IGF2BP2* genes, we found two low-frequency novel variants (MAF = 1.2% and 1.9% for *IGF2BP2* and *ARAP1*, respectively) showing independent association with insulin secretion adjusted for insulin sensitivity. Importantly, here, we demonstrated the association and measured the effect of each of the newly discovered low-frequency variants, both separately and analyzed together. Thus, in our southern European real-life cohort, we confirmed the role of the *ARAP1* and *IGF2BP2* genes in modulating insulin secretion assessed with DI. Further and deeper genetic studies are warranted to assess the presence and effects of low-frequency variants involved in insulin secretion.

## Figures and Tables

**Table 1:** Clinical features of all 757 participants showed 80% versus 20% (1st vs. 5th quintile) of DI distribution.

20% DI (*n* = 377)	Median	25° %	75° %	*p*	Median	25° %	75° %	80% DI (*n* = 380)
**Age (y)**	47	38	53.75	1.42 × 10^−23^	35	27	44.75	**Age**
**BMI (kg/m^2^)**	41.6	37.2	48.05	3.97 × 10^−10^	38	32.975	43.85	**BMI**
**Weight (kg)**	113.8	102.2	132.6	n.s.	108.5	96.48	129.35	**Weight**
**SBP (mmHg)**	130	120	140	5.07 × 10^−11^	120	110	130	**SBP**
**DBP (mmHg)**	80	75	90	0.0002	80	70	85	**DBP**
**FAT%**	47.5	41.9	51	0.01	46.25	40.15	49.325	**FAT%**
**Col (mg/dL)**	206	182	234.7	4.04 × 10^−7^	194	163.9	218	**Col**
**HDL (mg/dL)**	47	39	56	0.0002	50.05	42.1	60.48	**HDLc**
**LDL (mg/dL)**	128	108.99	152	0.000001	117	95.59	138.65	**LDLc**
**TG (mg/dL)**	134	96.4	179	2.30 × 10^−14^	99.75	74.43	134.33	**TRIG**
**AST (IU/L)**	21	17	28	0.00001	19	15	24	**AST**
**ALT (IU/L)**	26.9	20	40	3.50 × 10^−7^	22	16	32.63	**ALT**
**FPG 0′ (mg/dL)**	102	92	112	1.86 × 10^−67^	83	79	88	**OGTT 0′**
**Glu 120′ (mg/dL)**	14 4	115	176	1.63 × 10^−59^	99	84	114	**OGTT 120′**
**FPI 0′ (μIU/mL)**	27.35	16	43.8	7.87 × 10^−36^	12.6	7.7	20.2	**INS 0′**
**Ins 120′ (μIU/mL)**	119.35	62.025	199.25	1.34 × 10^−22^	57.45	28.45	96.98	**INS 120′**
**ISI**	0.48	0.28	0.87	4.25 × 10^−29^	1.04	0.65	1.80	**ISI**
**IGI**	0.27	0.02	0.60	1.52 × 10^−89^	2.20	1.28	3.59	**IGI**
**DI**	0.00223	0.00184	0.00275	1.86 × 10^−67^	0.00339	0.00301	0.00376	**DI**
**HOMA-IR**	7.10	4.10	11.49	1.70 × 10^−44^	2.64	1.67	4.42	**HOMA-IR**
**HOMA-B**	310.04	190.08	527.24	n.s.	299.14	169.97	465.47	**HOMA-B**
**c-pep 0’**	4.49	3.41	5.83	4.40 × 10^−17^	2.99	2.2975	3.87	**c-pep 0’**
**HbA1c (%)**	5.7	5.4	6.1	2.22 × 10^−31^	5.2	4.9	5.4	**HbA1c**

**Table 2:** Variant distribution by function and gene localization.

	ADAMTS9	ADYC5	IGF2BP2	CDKAL1	JAZF1	GCK	NAT2	KCNQ1	ARAP1	Total
**Variants**	311	171	89	95	23	91	23	113	268	1221
**Missense**	85	47	22	21	6	19	11	32	87	330
**Nonsense**	3	1	1	1	0	0	1	2	0	9
**Splicing**	6	1	0	1	0	0	0	5	2	15
**Frameshift**	51	24	10	1	0	0	0	5	2	93
**LoF**	145	73	33	24	6	19	12	44	91	447
**Synonymous**	85	47	22	21	8	19	11	34	87	334
**rs (known)**	83	50	24	27	4	25	6	45	54	325
**LoF/TOT**	47%	43%	37%	25%	26%	21%	52%	39%	34%	37%

LoF: loss of function, meaning missense, nonsense, splicing and frameshift variants; LoF/TOT: ratio between observed LoF and total variants; rs describes known variants. Total column is not the raw sum of single-gene data, as several variants were found in adjacent, intronic noncoding regions or in the antisense transcript.

**Table 3:** Binary logistic analysis of single variants associated with the lower extreme of DI, adjusted for age, gender and BMI.

Gene	Variant		*p*	O.R.	95% C.I.		Obs MAF
**IGF2BP2**	chr3:185363420 A > G	c.1708-9 A > G	0.003	0.142	0.039	0.527	1.19%
**GCK**	chr7:44186286 GA > G	c.864-70delA	0.014	0.384	0.179	0.825	2.51%
**ARAP1**	chr11:72422158 A > C	c.1121T > G; p.Val374Gly	0.011	0.304	0.121	0.763	1.92%

Only significant associations are shown. OR: odds ratio; CI: confidence interval; Obs MAF: observed minor allele frequency in our cohort.

**Table 4:** Binary logistic regression of significant variants associated with the lower extreme of DI as genetic risk model, adjusted for age, gender and BMI.

Gene	*p*	O.R	95% C.I.		R^2^ Nagelkerke	Sign H-L
**ARAP + IGF2BP2**	0.000093	0.223	0.105	0.473	0.254	0.015

OR: odds ratio; CI: confidence interval; Sign H-L: significance in Hosmer–Lemeshow test.

## Data Availability

The data presented in this study are available on reasonable request. The data are not publicly available due to privacy restrictions, lacking specific patients’ consent.

## Supplementary Materials

The following are available online: https://www.mdpi.com/article/10.3390/ijms23031221/s1.

## References

[1] International Diabetes Federation (2019). IDF Diabetes Atlas.

[2] Diabetes Care American Diabetes Association (2021). Standards of Medical Care in Diabetes, 2021. Diabetes Care.

[3] Stumvoll M., Goldstein B.J., van Haeften T.W. (2008). Type 2 diabetes: Pathogenesis and treatment. Lancet.

[4] Laakso M. (2019). Biomarkers for type 2 diabetes. Mol. Metab..

[5] Tremblay J., Hamet P. (2019). Metabolism. Environmental and genetic contributions to diabetes. Metabolism.

[6] Udler M.S., Kim J., von Grotthuss M., Bonàs-Guarch S., Cole J.B., Chiou J., Christopher D., Boehnke M., Laakso M., Anderson on behalf of METASTROKE and the ISGC (2018). Type 2 diabetes genetic loci informed by multi-trait associations point to disease mechanisms and subtypes: A soft clustering analysis. PLoS Med..

[7] Udler M.S. (2019). Type 2 Diabetes: Multiple Genes, Multiple Diseases. Curr. Diab. Rep..

[8] Găman M.A., Epîngeac M.E., Diaconu C.C., Găman A.M. (2020). Evaluation of oxidative stress levels in obesity and diabetes by the free oxygen radical test and free oxygen radical defence assays and correlations with anthropometric and laboratory parameters. World J. Diabetes.

[9] Cozma M.A., Găman M.A., Dobrică E.C., Boroghină S.C., Iancu M.A., Crețoiu S.M., Simionescu A.A. (2021). A Glimpse at the Size of the Fetal Liver-Is It Connected with the Evolution of Gestational Diabetes?. Int. J. Mol. Sci..

[10] Ahlqvist E., Storm P., Käräjämäki A., Martinell M., Dorkhan M., Carlsson A., Vikman P., Prasad R.B., Aly D.M., Almgren P. (2018). Novel subgroups of adult-onset diabetes and their association with outcomes: A data-driven cluster analysis of six variables. Lancet Diabetes Endocrinol..

[11] Dennis J.M., Shields B.M., Henley W.E., Jones A.G., Hattersley A.T. (2019). Disease progression and treatment response in data-driven subgroups of type 2 diabetes compared with models based on simple clinical features: An analysis using clinical trial data. Lancet Diabetes Endocrinol..

[12] Mahajan A., Taliun D., Thurner M., Robertson N.R., Torres J.M., Rayner N.W., Payne A.J., Steinthorsdottir V., Scott R.A., Grarup N. (2018). Fine-mapping type 2 diabetes loci to single-variant resolution using high-density imputation and islet-specific epigenome maps. Nat. Genet..

[13] Suzuki K., Akiyama M., Ishigaki K., Kanai M., Hosoe J., Shojima N., Hozawa A., Kadota A., Kuriki K., Naito M. (2019). Identification of 28 new susceptibility loci for type 2 diabetes in the Japanese population. Nat. Genet..

[14] Manolio T.A., Collins F.S., Cox N.J., Goldstein D.B., Hindorff L.A., Hunter D.J., McCarthy M.I., Ramos E.M., Cardon L.R., Chakravarti A. (2009). Finding the missing heritability of complex diseases. Nature.

[15] Génin E. (2020). Missing heritability of complex diseases: Case solved?. Hum. Genet..

[16] Meigs J.B. (2019). The Genetic Epidemiology of Type 2 Diabetes: Opportunities for Health Translation. Curr. Diab. Rep..

[17] Mohlke K.L., Boehnke M. (2015). Recent advances in understanding the genetic architecture of type 2 diabetes. Hum. Mol. Genet..

[18] Scott R.A., Scott L.J., Mägi R., Marullo L., Gaulton K.J., Kaakinen M., Pervjakova N., Pers T.H., Johnson A.D., Eicher J.D. (2017). An Expanded Genome-Wide Association Study of Type 2 Diabetes in Europeans. Diabetes.

[19] Bonàs-Guarch S., Guindo-Martínez M., Miguel-Escalada I., Grarup N., Sebastian D., Rodriguez-Fos E., Sánchez F., Planas-Fèlix M., Cortes-Sánchez P., González S. (2018). Re-analysis of public genetic data reveals a rare X-chromosomal variant associated with type 2 diabetes. Nat. Commun..

[20] Gaulton K.J., Ferreira T., Lee Y., Raimondo A., Mägi R., Reschen M.E., Mahajan A., Locke A., Rayner N.W., Robertson N. (2015). Genetic fine mapping and genomic annotation defines causal mechanisms at type 2 diabetes susceptibility loci. Nat. Genet..

[21] Mahajan A., Wessel J., Willems S.M., Zhao W., Robertson N.R., Chu A.Y., Gan W., Kitajima H., Taliun D., Rayner N.W. (2018). Refining the accuracy of validated target identification through coding variant fine-mapping in type 2 diabetes. Nat. Genet..

[22] Rivas M.A., Beaudoin M., Gardet A., Stevens C., Sharma Y., Zhang C.K., Boucher G., Ripke S., Ellinghaus D., Burtt N. (2011). Deep resequencing of GWAS loci identifies independent rare variants associated with inflammatory bowel disease. Nat. Genet..

[23] Khetarpal S.A., Babb P.L., Zhao W., Hancock-Cerutti W.F., Brown C.D., Rader D.J., Voight B.F. (2018). Multiplexed Targeted Resequencing Identifies Coding and Regulatory Variation Underlying Phenotypic Extremes of High-Density Lipoprotein Cholesterol in Humans. Circ. Genom. Precis. Med..

[24] Cohen J.C., Kiss R.S., Pertsemlidis A., Marcel Y.L., McPherson R., Hobbs H.H. (2004). Multiple rare alleles contribute to low plasma levels of HDL cholesterol. Science.

[25] Bonnefond A., Froguel P. (2015). Rare and common genetic events in type 2 diabetes: What should biologists know?. Cell Metab..

[26] Sidore C., Busonero F., Maschio A., Porcu E., Naitza S., Zoledziewska M., Mulas A., Pistis G., Steri M., Danjou F. (2015). Genome sequencing elucidates Sardinian genetic architecture and augments association analyses for lipid and blood inflammatory markers. Nat. Genet..

[27] Lee S., Abecasis G.R., Boehnke M., Lin X. (2014). Rare-variant association analysis: Study designs and statistical tests. Am. J. Hum. Genet..

[28] Peloso G.M., Rader D.J., Gabriel S., Kathiresan S., Daly M.J., Neale B.M. (2016). Phenotypic extremes in rare variant study designs. Eur. J. Hum. Genet..

[29] Guey L.T., Kravic J., Melander O., Burtt N.P., Laramie J.M., Lyssenko V., Jonsson A., Lindholm E., Tuomi T., Isomaa B. (2011). Power in the phenotypic extremes: A simulation study of power in discovery and replication of rare variants. Genet. Epidemiol..

[30] Romeo S., Pennacchio L.A., Fu Y., Boerwinkle E., Tybjaerg-Hansen A., Hobbs H.H., Cohen J.C. (2007). Population-based resequencing of ANGPTL4 uncovers variations that reduce triglycerides and increase HDL. Nat. Genet..

[31] Heid I.M., Huth C., Loos R.J., Kronenberg F., Adamkova V., Anand S.S., Ardlie K., Biebermann H., Bjerregaard P., Boeing H. (2009). Meta-analysis of the INSIG2 association with obesity including 74,345 individuals: Does heterogeneity of estimates relate to study design?. PLoS Genet..

[32] Cobelli C., Toffolo G.M., Dalla Man C., Campioni M., Denti P., Caumo A., Butler P., Rizza R. (2007). Assessment of beta-cell function in humans, simultaneously with insulin sensitivity and hepatic extraction, from intravenous and oral glucose tests. Am. J. Physiol. Endocrinol. Metab..

[33] DeFronzo R.A., Banerji M.A., Bray G.A., Buchanan T.A., Clement S., Henry R.R., Kitabchi A.E., Mudaliar S., Musi N., Ratner R. (2010). Determinants of glucose tolerance in impaired glucose tolerance at baseline in the Actos Now for Prevention of Diabetes (ACT NOW) study. Diabetologia.

[34] Bergman R.N., Ader M., Huecking K., Van Citters G. (2002). Accurate assessment of beta-cell function: The hyperbolic correction. Diabetes.

[35] Cobelli C., Dalla Man C., Toffolo G., Basu R., Vella A., Rizza R. (2014). The oral minimal model method. Diabetes.

[36] Ferrannini E., Mari A. (2014). β-Cell function in type 2 diabetes. Metabolism.

[37] Imamura M., Maeda S. (2011). Genetics of type 2 diabetes: The GWAS era and future perspectives [Review]. Endocr. J..

[38] Knowles J.W., Xie W., Zhang Z., Chennamsetty I., Assimes T.L., Paananen J., Hansson O., Pankow J., Goodarzi M.O., Carcamo-Orive I. (2015). Identification and validation of N-acetyltransferase 2 as an insulin sensitivity gene. J. Clin. Investig..

[39] Cao J., Mu Q., Huang H. (2018). The Roles of Insulin-Like Growth Factor 2 mRNA-Binding Protein 2 in Cancer and Cancer Stem Cells. Stem. Cells Int..

[40] Grarup N., Sparsø T., Hansen T. (2010). Physiologic characterization of type 2 diabetes-related loci. Curr. Diab. Rep..

[41] Christiansen J., Kolte A.M., Hansen T., Nielsen F.C. (2009). IGF2 mRNA-binding protein 2: Biological function and putative role in type 2 diabetes. J. Mol. Endocrinol..

[42] Schaeffer V., Hansen K.M., Morris D.R., LeBoeuf R.C., Abrass C.K. (2012). RNA-binding protein IGF2BP2/IMP2 is required for laminin-β2 mRNA translation and is modulated by glucose concentration. Am. J. Physiol. Renal. Physiol..

[43] Rodriguez S., Eiriksdottir G., Gaunt T.R., Harris T.B., Launer L.J., Gudnason V., Day I.N. (2010). IGF2BP1, IGF2BP2 and IGF2BP3 genotype, haplotype and genetic model studies in metabolic syndrome traits and diabetes. Growth Horm. IGF Res..

[44] Dai N., Zhao L., Wrighting D., Krämer D., Majithia A., Wang Y., Cracan V., Borges-Rivera D., Mootha V.K., Nahrendorf M. (2015). IGF2BP2/IMP2-Deficient mice resist obesity through enhanced translation of Ucp1 mRNA and Other mRNAs encoding mitochondrial proteins. Cell Metab..

[45] Livingstone C., Borai A. (2014). Insulin-like growth factor-II: Its role in metabolic and endocrine disease. Clin. Endocrinol..

[46] Sharma S., Mathre S., Ramya V., Shinde D., Raghu P. (2019). Phosphatidylinositol 5 Phosphate 4-Kinase Regulates Plasma-Membrane PIP3 Turnover and Insulin Signaling. Cell Rep..

[47] Kulzer J.R., Stitzel M.L., Morken M.A., Huyghe J.R., Fuchsberger C., Kuusisto J., Laakso M., Boehnke M., Collins F.S., Mohlke K.L. (2014). A common functional regulatory variant at a type 2 diabetes locus upregulates ARAP1 expression in the pancreatic beta cell. Am. J. Hum. Genet..

[48] Strawbridge R.J., Dupuis J., Prokopenko I., Barker A., Ahlqvist E., Rybin D., Petrie J.R., Travers M.E., Bouatia-Naji N., Dimas A.S. (2011). Genome-wide association identifies nine common variants associated with fasting proinsulin levels and provides new insights into the pathophysiology of type 2 diabetes. Diabetes.

[49] Sztul E., Chen P.W., Casanova J.E., Cherfils J., Dacks J.B., Lambright D.G., Lee F.S., Randazzo P.A., Santy L.C., Schürmann A. (2019). ARF GTPases and their GEFs and GAPs: Concepts and challenges. Mol. Biol. Cell.

[50] Lawrence J.T., Birnbaum M.J. (2003). ADP-ribosylation factor 6 regulates insulin secretion through plasma membrane phosphatidylinositol 4,5-bisphosphate. Proc. Natl. Acad. Sci. USA.

[51] Cuthbert E.J., Davis K.K., Casanova J.E. (2008). Substrate specificities and activities of AZAP family Arf GAPs in vivo. Am. J. Physiol. Cell Physiol..

[52] Carrat G.R., Hu M., Nguyen-Tu M.S., Chabosseau P., Gaulton K.J., van de Bunt M., Siddiq A., Falchi M., Thurner M., Canouil M. (2017). Decreased STARD10 Expression Is Associated with Defective Insulin Secretion in Humans and Mice. Am. J. Hum. Genet..

[53] Jayaram B., Syed I., Kyathanahalli C.N., Rhodes C.J., Kowluru A. (2011). Arf nucleotide binding site opener [ARNO] promotes sequential activation of Arf6, Cdc42 and Rac1 and insulin secretion in INS 832/13 β-cells and rat islets. Biochem. Pharmacol..

[54] Nejentsev S., Walker N., Riches D., Egholm M., Todd J.A. (2009). Rare variants of IFIH1, a gene implicated in antiviral responses, protect against type 1 diabetes. Science.

[55] Abdul-Ghani M.A., Matsuda M., Balas B., DeFronzo R.A. (2007). Muscle and liver insulin resistance indexes derived from the oral glucose tolerance test. Diabetes Care.

[56] Matsuda M., DeFronzo R.A. (1999). Insulin sensitivity indices obtained from oral glucose tolerance testing: Comparison with the euglycemic insulin clamp. Diabetes Care.

[57] Cersosimo E., Solis-Herrera C., Trautmann M.E., Malloy J., Triplitt C.L. (2014). Assessment of pancreatic β-cell function: Review of methods and clinical applications. Curr. Diabetes Rev..

[58] Danecek P., Bonfield J.K., Liddle J., Marshall J., Ohan V., Pollard M.O., Whitwham A., Keane T., McCarthy S.A., Davies R.M. (2021). Twelve years of SAMtools and BCFtools. Gigascience.

[59] Richards S., Aziz N., Bale S., Bick D., Das S., Gastier-Foster J., Grody W.W., Hegde M., Lyon E., Spector E. (2015). Standards and guidelines for the interpretation of sequence variants: A joint consensus recommendation of the American College of Medical Genetics and Genomics and the Association for Molecular Pathology. Genet. Med..

[60] Bewick V., Cheek L., Ball J. (2005). Statistics review 14: Logistic regression. Crit. Care.

