# Supplementary material for: Deep Resequencing of 9 Candidate Genes Identifies a Role for ARAP1 and IGF2BP2 in Modulating Insulin Secretion Adjusted for Insulin Resistance in Obese Southern Europeans
Source: Int J Mol Sci. 2022 Jan 22;23(3):1221. doi: 10.3390/ijms23031221 (PMC8835579; doi:10.3390/ijms23031221)
Supplement: Supplementary file 1 [file ijms-23-01221-s001.zip › ijms-1530505-supplementary.pdf]

Deep Resequencing of 9 candidate genes identifies a role for ARAP1 and IGF2BP2 in modulating insulin secretion adjusted for insulin resistance in obese Southern Europeans

Supplmentary Tables

Supplementary Table S1. Selected genes for genetic and related published O.R.

Gene	O.R.	95% C.I.
**ADAMTS9**	1.09	1.06-1.12
**ADCY5**	1.12	1.09-1.15
**CDAKL1**	1.12	1.08-1.16
**IGF2BP2**	1.17	1.10-1.25
**JAZF1**	1.10	1.07-1.13
**GCK**	1.07	1.05-1.10
**NAT2**	0.35	0.25–0.49
**KCNQ1**	1.41	1.29-1.55
**ARAP1**	1.14	1.11-1.17

From: 1 Imamura M Endocrine Journal 2011; 2 Knowles Jw Et Al. J Clin Invest 2015

Supplementary Table S2. Binary Logistic Regression with norm-glycaemic status associate with carrying any of two selected variants of genetic-risk model (GRM2), adjusted for age, gender and BMI

	p	O.R.	95% C.I.		R^2 Negelkerke	p H-L
NGT	0,022	2,411	1,136	5,116	0,245	0,042

NGT: Normal Glucose Tolerance, versus Non-normal glucose tolerance (IFG, IGT and T2D), according to ADA 2021. O.R.: Odd Ratio; C.I.: Confidence Interval; p H-L: significance in Hosmer–Lemeshow test.

Supplementary References

1. Imamura M, Maeda S. Genetics of type 2 diabetes: the GWAS era and future perspectives [Review]. *Endocr J.* **2011** ;58(9):723-39. doi: 10.1507/endocrj.ej11-0113.
2. Knowles JW, Xie W, Zhang Z, Chennamsetty I, Assimes TL, Paananen J, Hansson O, Pankow J, Goodarzi MO, Carcamo-Orive I, Morris AP, Chen YD, Mäkinen VP, Ganna A, Mahajan A, Guo X, Abbasi F, Greenawalt DM, Lum P, Molony C, Lind L, Lindgren C, Raffel LJ, Tsao PS; RISC (Relationship between Insulin Sensitivity and Cardiovascular Disease) Consortium; EUGENE2 (European Network on Functional Genomics of Type 2 Diabetes) Study; GUARDIAN (Genetics UndeRlying DIAbetes in HispaNics) Consortium; SAPPHIRe (Stanford Asian and Pacific Program for Hypertension and Insulin Resistance) Study, Schadt EE, Rotter JI, Sinaiko A, Reaven G, Yang X, Hsiung CA, Groop L, Cordell HJ, Laakso M, Hao K, Ingelsson E, Frayling TM, Weedon MN, Walker M, Quertermous T. Identification and validation of N-acetyltransferase 2 as an insulin sensitivity gene. *J Clin Invest*. **2015** Apr;125(4):1739-51. doi: 10.1172/JCI74692. Erratum in: J Clin Invest. 2016 Jan;126(1):403. Chennemsetty, Indumathi [corrected to Chennamsetty, Indumathi].
